# Insole Systems for Disease Diagnosis and Rehabilitation: A Review

**DOI:** 10.3390/bios13080833

**Published:** 2023-08-21

**Authors:** Zhiyuan Zhang, Yanning Dai, Zhenyu Xu, Nicolas Grimaldi, Jiamu Wang, Mufan Zhao, Ruilin Pang, Yueming Sun, Shuo Gao, Hu Boyi

**Affiliations:** 1 School of Instrumentation and Optoelectronic Engineering, Beihang University, Beijing 100191, China; exzzy32@163.com (Z.Z.); 16171056@buaa.edu.cn (Y.D.); 20373962@buaa.edu.cn (Z.X.); 2 J. Crayton Pruitt Family Department of Biomedical Engineering, University of Florida, Gainesville, FL 32611, USA; n.grimaldi@ufl.edu; 3 School of Transportation Science and Engineering, Beihang University, Beijing 100191, China; 20376276@buaa.edu.cn; 4 School of Artificial Intelligence, Beihang University, Beijing 100191, China; 20373270@buaa.edu.cn; 5 School of Automation Science and Electrical Engineering, Beihang University, Beijing 100191, China; 20373816@buaa.edu.cn; 6 School of Electronics and Information Engineering, Beihang University, Beijing 100191, China; 20373180@buaa.edu.cn; 7 School of Industrial and Systems Engineering, University of Florida, Gaineville, FL 32611, USA

**Keywords:** gait analysis, chronic diseases, plantar pressure, insole systems

## Abstract

Some chronic diseases, including Parkinson’s disease (PD), diabetic foot, flat foot, stroke, elderly falling, and knee osteoarthritis (KOA), are related to orthopedic organs, nerves, and muscles. The interaction of these three parts will generate a comprehensive result: gait. Furthermore, the lesions in these regions can produce abnormal gait features. Therefore, monitoring the gait features can assist medical professionals in the diagnosis and analysis of these diseases. Nowadays, various insole systems based on different sensing techniques have been developed to monitor gait and aid in medical research. Hence, a detailed review of insole systems and their applications in disease management can greatly benefit researchers working in the field of medical engineering. This essay is composed of the following sections: the essay firstly provides an overview of the sensing mechanisms and parameters of typical insole systems based on different sensing techniques. Then this essay respectively discusses the three stages of gait parameters pre-processing, respectively: pressure reconstruction, feature extraction, and data normalization. Then, the relationship between gait features and pathogenic mechanisms is discussed, along with the introduction of insole systems that aid in medical research; Finally, the current challenges and future trends in the development of insole systems are discussed.

## 1. Introduction

The branches of chronic diseases are various and include neurologic, orthopedic, and musculoskeletal diseases. Their clinical manifestations, such as motor impairment and tissue damage, can lead to death and disability in older people [1]. The features of these diseases can include being asymptomatic, having long incubation periods, involving multiple pathogens, and causing numerous complications and related dysfunctions. Therefore, effective methods of diagnosis and rehabilitation are in high demand. Conventionally, rehabilitation and diagnosis are mainly conducted in hospitals, where medical professionals provide appropriate prescriptions supported by methods such as medical assays and X-ray films. However, with the rapid development of an aging society, the number of orthopedic and neurologic chronic patients is increasing, which places heavy burdens on medical institutions and staff [2]. As a result, the demand for more flexible and remote medical methods has grown. In pursuit of this goal, novel recovery methods based on the Internet of Healthcare Things (IoHT) have emerged in the past few decades. IoHT refers to the application of network systems to connect patients and their medical data with available healthcare resources such as hospitals, doctors, and nurses [2,3]. The development of IoHT has led to the creation of wearable medical devices, which have made significant contributions to both medical professionals and patients. For medical professionals, these devices allow them to monitor the human body and remotely evaluate health status while patients are using them [4]. As for patients, they can seamlessly collect data during their daily activities. The collection of data on specific regions of the body does not pose a risk of privacy leakage [5]. Additionally, thanks to the small size and lightweight nature of wearable sensors, patients will experience minimal disruption most of the time [4].

Among various wearable devices, insole systems are particularly suitable for analyzing diseases. The most significant reason is that neurologic chronic diseases, such as Parkinson’s disease (PD) and cerebrovascular accidents (strokes), are caused by lesions in the central nervous system (CNS) and peripheral nerves (PN) [6]. Additionally, typical orthopedic diseases, like flat feet, are closely associated with lower limb lesions [6]. Given these characteristics, gait features have the ability to comprehensively reflect these physiological systems [7]. Therefore, by analyzing these gait patterns, related results such as plantar pressure distribution can contribute to the monitoring of orthopedic and neurologic lesions, such as foot ulceration and loss of dopamine [8], and provide valuable information for recovery.

**Figure 1:**
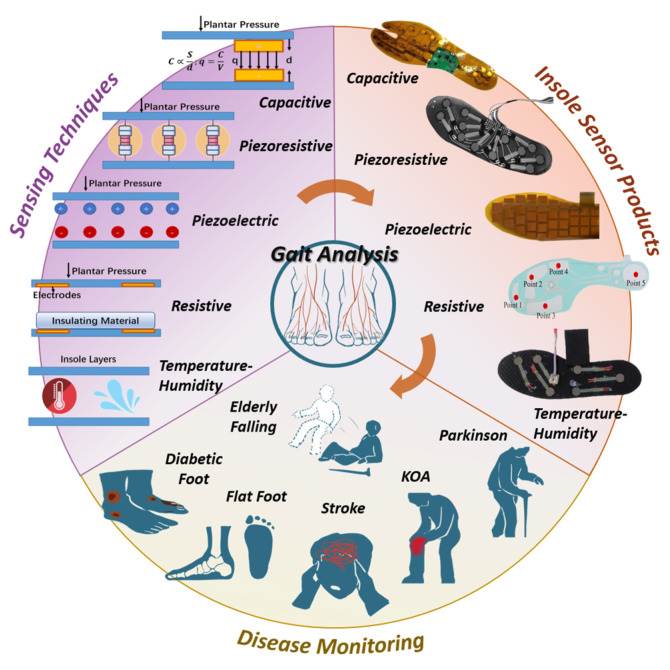
Demonstration of diseases, sensing techniques and current products. Sensing techniques are the foundation of insole systems, while insole systems are used to assist the diagnosis and rehabilitation of diseases.

During the medical application of these sensors (as shown in Figure 1), selecting appropriate techniques and systems for specific diseases is crucial, particularly when the product is intended for assisting in diagnosis. Therefore, conducting an in-depth review of the essential pathogenic mechanisms, sensing techniques, current products, and the correlation between diseases and gait features is highly necessary. However, previous essays have not yet provided a specific focus on these aspects, as this field is highly interdisciplinary. Hence, this article is presented with the aim of addressing this gap.

The article aims to review the mechanisms and applications of the insole plantar pressure sensor systems for disease detection, and the pre- and post-processing techniques used to analyze the data or indicators to be measured in relation to a given disease.

Hence, compared to previous and present works, this essay specifically contributes to the following aspects:

- Provides an overview of current commercial and institutional insole systems, presenting their parameters, merits, and drawbacks.
- Establishes the connection between pathogenic mechanisms and abnormal features of six diseases.
- Conducts a detailed review of how insole systems have assisted in the diagnosis and rehabilitation of each disease.

To conduct this work, we searched references from IEEE, Wiley, Web of Science, and other sources. Key words included ‘neurological and orthopedic diseases’, ‘insole systems’, ‘plantar pressure sensors’, and so on. Based on different research focuses, the collected references were categorized into three aspects. Theoretical medical essays provided us with information on abnormal gait features, such as a long stance phase and reduced peak pressure. Research papers on current plantar pressure sensors offered insights into the mechanisms and parameters of common sensing techniques. Additionally, research articles on medical applications provided valuable information on how insole systems are utilized to assist in medical analysis.

**Figure 2:**
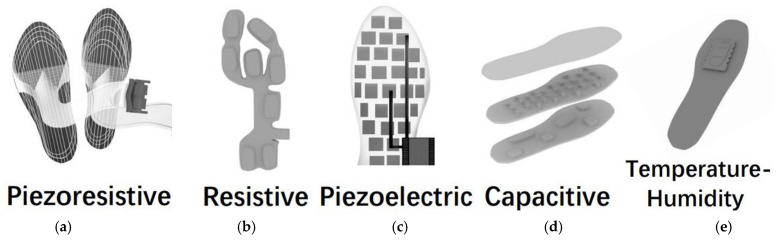
Illustration of prototypes of (**a**) piezoresistive, (**b**) resistive, (**c**) piezoelectric, (**d**) capacitive and (**e**) temperature-humidity insole sensing techniques. These figures are inspired by references [10,14,18,19,20,32] but are originally drawn by authors of this article.

In Section 2, we classified current products according to sensing techniques and summarized their principles and parameters based on the literature. Additionally, we provide definitions of terms commonly used in gait monitoring procedures, as depicted in Figure 2.

**Figure 3:**
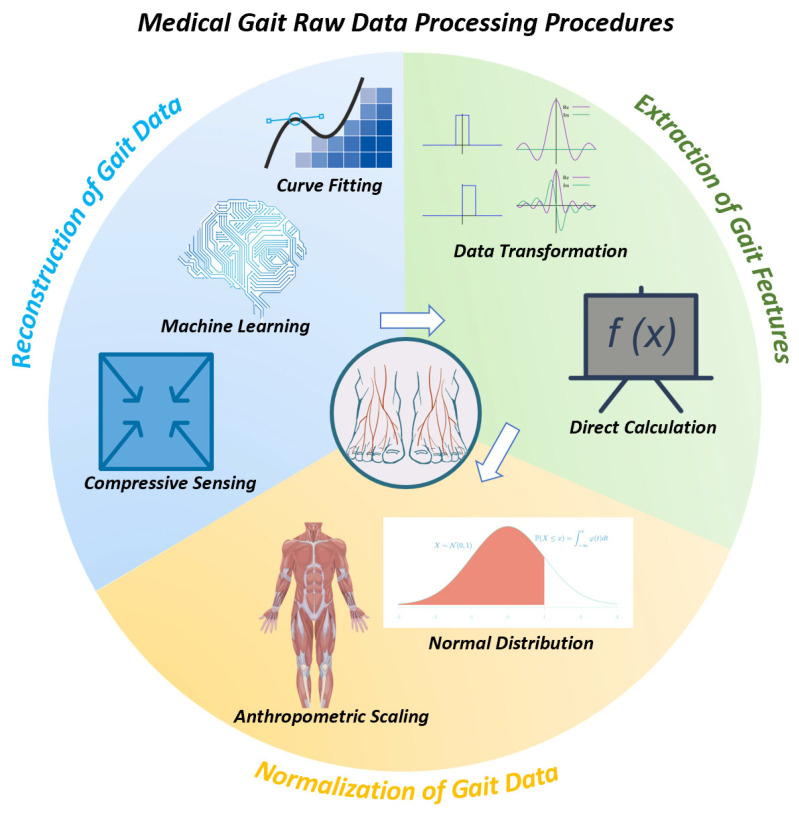
Three steps of processing gait data for medical purposes: 1. Reconstruction, 2. Extraction, 3. normalization. Suitable techniques of each step are mentioned in Section 3, Section 4 and Section 5.

In Section 3, we firstly present the three stages of data processing for gait data before medical application, namely reconstruction, extraction, and normalization (as shown in Figure 3). Then, we introduce three frequently used methods and their applications in the reconstruction stage: machine learning, compressive sensing, and fitting.

In Section 4, we further specify the definition and significance of feature extraction. We introduce the main techniques used for feature extraction, including direct calculation and data transformation, along with relevant examples.

In Section 5, we emphasize the individual differences present in gait data, highlighting the importance of normalization to eliminate these differences. We then review two useful methods for normalization: data transformation and anthropometric scaling. The significant results of anthropometric scaling are presented in Table 3 along with their explanations.

In Section 6, we conduct a comprehensive review of six typical chronic diseases and their corresponding insole systems, considering three key aspects: pathogenic principles, abnormal gait features, and the medical assistance provided by insole systems. Detailed diagrams are included to enhance the specificity of the review. By analyzing the gathered information, we summarize the designing methodology of insole systems for each disease.

In Section 7, we explore the black-box issue and the inherent features of gait that have resulted in reduced interpretability and accuracy, thereby imposing limitations on current insole systems. Consequently, taking into account potential trends and advancements in medical-assisted devices, we propose that the utilization of multi-sensing-based digital twin models and predictions for various living scenarios can enhance the performance of future insole systems.

## 2. Literature Review about Mainstream Detecting Methods

Insole sensors are designed with the purpose of measuring temporal and spatial gait parameters such as cycle time, swing and stance phase, step and stride length, and pressure distribution [9]. Traditionally, these objectives are achieved through plantar force sensing mechanisms, which include piezoresistive, resistive, capacitive, piezoelectric, and temperature-humidity methods, as illustrated in Figure 1.

In the following paragraphs, we provide an overview of seven distinct insole pressure sensing mechanisms and their corresponding current products.

### 2.1. Piezoresistive Technique

The principle of this technique revolves around establishing relationships between applied stress and the resulting change in the electric resistance of a material. Plantar stress detection can be achieved through two conventional methods: the adhesive method and screen-printing technology.

The adhesive method involves attaching individual force-sensing resistors (FSRs) to specific regions of the insole based on the symptoms of diseases. For example, the insole system for Parkinson’s disease (PD) includes 16 FSRs positioned at the forefoot and heel [10]. Additionally, the system is enclosed in shrink-wrap to prevent displacement and minimize motion-induced abrasion.

Drawbacks of adhesive insole systems, such as sensor overlap, friction, and unstable mechanical capacity, can be addressed through the use of a whole-piece design and fabrication techniques based on screen printing technology. As a result, several commercialized products have emerged, including the F-scan^®^ system [11] which offers a sensing pressure range of 7 kPa to 1043 kPa; the sensor array based on multi-walled carbon nanotube-polydimethylsiloxane [12]; and the SurroSense Rx Insole developed by Orpyx SI. Further advancements in this field are focused on shear stress detection. One proposed system is capable of measuring normal stress up to 400 kPa and shear stress up to 80 kPa [13].

The virtues of piezoresistive sensors, such as high voltage sensitivity and affordability, enable their diverse applications. However, issues like hysteresis and creep may arise during their usage, necessitating frequent calibration. As a result, this technique is more suitable for short-term diseases rather than long-lasting therapy and monitoring.

### 2.2. Resistive Techniques

These techniques are accomplished by establishing relationships between resistance, element geometry, and structure. Traditionally, resistive force sensing can be achieved through contact surfaces and strain gauges.

The former method utilizes two resistive layers isolated by bracing frames, the imposed load will extrude them to approach. When in contact, current flows through the layers. The contacted area, which is correlated to an external force, can be reflected in the resistance. In a previous study [14], an insole system for diabetic patients was presented, allowing real-time tracking. The contact area between layers increases as the applied force increases.

The latter method involves attaching strain gauges to mechanical structures, which measure the strain when pressure is applied. The strain in the resistive material results in a change in resistance. For example, a previous study [15] proposed a mechanical insole comprised of strain gauge load cells and multiple-axis force sensors. This system can provide synchronous measurements of shear load and normal load.

The Tek-scan company produces highly-integrated resistive and piezoresistive insole systems. These systems offer long-term tracking capabilities due to their low unrepeatability [14] and affordability. However, it is important to note that these systems do not have simple structures, which can result in larger volumes. Additionally, their intricate structure may lead to durability issues, potentially reducing their user base over time.

### 2.3. Capacitive Techniques

As capacitance is significantly influenced by the dielectric constant, capacitive sensors are sensitive to environmental humidity and electromagnetic interference. Typically, capacitive sensors are constructed with an elastomer medium and two electrode layers. The applied load affects the capacitance by altering the distance between the upper and lower layers, allowing plantar stress to be determined through capacitance measurements. Current products in this category can be classified into experimental systems and commercial systems. An example of an experimental study presents an integrative method [16]. This work involves the utilization of sensor layers with fixed capacitance arrays, along with a flexible circuit layer integrated into the system. This particular system is well-suited for monitoring the rehabilitation progress of stroke patients.

Commercial capacitive insole products have similar advantages with piezoelectric plantar sensors, the common advantage prioritize durability and long-term utilization [17,18,19,20,21,22,23,24], which making them suitable for patient tracking. One existing product boasts a prolonged working lifetime, capable of supporting 100 km of running. It can measure extreme pressures up to 400 kPa, making it suitable for examining weight-supporting gait patterns following extremity fractures [17]. Additionally, a commercial product based on Pedar-X [18] offers sustained and continuous utilization. A difference between piezoelectric PSD sensors and capacitive PSD sensors is the former type do not have much commercial products because piezoelectric PSD sensors are easily influenced by unstable experimental environment [19,20].

As a result, same advantages in long-time utilization make capacitive and piezoelectric plantar pressure sensors can be widely used in long time monitoring process of rehabilitation or diagnosis, such as knee operation [21] of diabetic foot ulcers [22,23] and ankle osteoarthritis (AO) [24], as these conditions require long-term data recording for predictive purposes. Moticon, a company producing open-go capacitive insole systems, also provides solutions for diabetic foot diagnosis and supervision.

Sustained working lifetime and dynamic performance are virtues of capacitive sensors, but the limited force sensing range should be considered when choosing this technique.

### 2.4. Piezoelectric Techniques

Piezoelectric techniques rely on the accumulation of electrical charges on the surface of non-centrally symmetric materials when an external force is applied [19,20,21]. In piezoelectric insole sensors, this technique is commonly implemented using electrode/piezoelectric film/electrode sandwiched structures. Piezoelectric sensors offer virtues such as high sensitivity, low power consumption, and a simple structure [19,20,21].

A specific prototype [25] has been designed for early-stage diabetes diagnosis. It utilizes eight separate sensors with rigid substrate structures to minimize the negative impacts of crosstalk from other axes. Additionally, the essay [23] demonstrates that flexible printed circuit boards (PCBs) serve as suitable electrode layers for mass production, because PCB boards can make piezoelectric sensors more stable.

Another advantage of piezoelectric sensors is their ability to detect forces in different directions. This is due to the fact that the piezoelectric effect can occur on both vertical and parallel axes to the film surface, and the force-to-voltage coefficient depends on the strength and polarization directions. Previous work [24] achieves this by utilizing a transducer alongside four independent piezoelectric film sensors.

However, due to the polarization orientation limitations of piezoelectric materials, it is challenging to align them in the same direction. This can result in responses from unwanted forces. Additionally, another drawback is the leakage generated in the subsequent amplifying circuit, which means that they cannot measure static forces. These reasons have contributed to the lack of commercialization of piezoelectric-based sensors.

### 2.5. Temperature and Humidity-Based Techniques

Patients with peripheral neuropathies are at an increased risk of lower extremity skin breakdown [26,27], which can be indicated by changes in temperature and moisture levels [28,29]. To detect inflammation and tissue damage caused by repetitive stress, medical professionals have proposed methods for measuring insole and skin temperature [30,31].

Previous methods for measuring insole temperature mainly relied on thermistors, where changes in resistance reflect temperature changes. For example, three thermistors with a resistance of 5 k are placed at the heel, big toe, and ball of the foot. However, the sensitivity of these methods is limited as they can only detect temperature differences greater than 2.2 °C [32].

To increase sensitivity, more integrated sensors have been developed in the last decade. The SHT series produced by Sensirion [33,34] and the DHT series produced by Aosong Electronics [35] are widely used temperature sensors. For instance, the SHT-21 module, fixed on a printed circuit board (PCB), achieves a sensitivity of 4% and 0.3 °C when the temperature fluctuation is less than 15 °C [31]. Similarly, the SHT-11 module, integrated with an amplifier and A/D converter in a compact chip, demonstrates a sensitivity of 3% and 0.4 °C [34]. These sensors are suitable for the diagnosis and monitoring of foot skin-related conditions, such as diabetic ulcers. A representative insole system produced by Orpyx can measure temperature and pressure simultaneously, thus providing more comprehensive data for diagnosis.

### 2.6. Virtues and Drawbacks of Techniques

The virtues and drawbacks of these techniques make them suitable for diverse diseases. Piezoresistive and resistive methods have a high force sensing range and low price, but they suffer from low repeatability and are influenced by temperature and humidity. Temperature- and humidity-based sensors have low energy consumption and cost, but their inability to detect plantar pressure limits their application in gait-related analysis. On the other hand, capacitance and piezoelectric methods offer high sensitivity to force, but they are also sensitive to temperature and electromagnetic interference changes.

To demonstrate the models more clearly, we present drafts of prototypes of five sensing techniques-based plantar pressure sensors systems. Hence, the Figure 2 [10,14,18,21,22,32] is present here. 

## 3. The Reconstruction of Plantar Pressure from Gait Data

The sensing techniques mentioned above provide effective methods for measuring gait parameters such as peak plantar pressure and step length. Afterwards, the raw data requires pre-processing before assisting medical diagnosis. Figure 3 is present to demonstrate the stages of Section 3, Section 4 and Section 5.

Generally, the process can be divided into three steps [36,37]: 1. Reconstruction of plantar stress distribution (PSD), 2. Extraction of gait features, 3. Normalization.

The rationale can be summarized as follows: 1. Raw data gathered from sensors can not be applied in medical scenarios directly, and reconstruction is necessary to extract desired information. 2. Gait features are variable; thus, the useful features need to be selected and extracted. 3. Individual differences in gait features can hinder the accuracy of diagnosis, thus requiring normalization [36,37].

**Figure 4:**
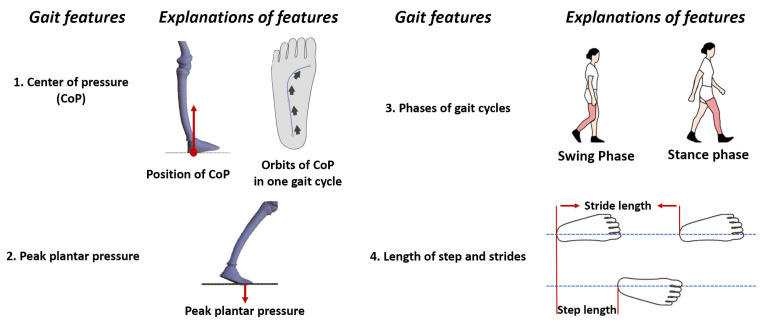
Illustrations of four predominant gait features, complementary features like gait velocity and pressure distribution can be obtained using the features above.

As shown in Figure 4, each step can be achieved through distinct reliable methods, each of which will be introduced separately in Section 3, Section 4 and Section 5.

The reconstruction of PSD can be succinctly described as converting analog signals into digital signals for further computer-based processing methods. The first step is to sample the plantar force signal, ensuring it satisfies the Nyquist sampling theorem. The resulting digital signal can be analyzed directly, or its spectrum can be rebuilt using Fourier transform for further analysis. Based on these principles, three current frequently used methods have been proposed and classified [37]: compressed sensing (CS), machine learning (ML), and fitting [37]. Table 1 [19,38,39,40,41,42,43,44,45,46] provides a brief detail of each example in Section 3.

### 3.1. Fitting

In its early stages, insole systems suffered from a limited number of sensors, leading to two problems: limited coverage and inadequate data for PSD [47]. The former was addressed by designing specific sensing points throughout the insole [48]. However, the latter proved to be a more complex problem, requiring the development of fitting methods. Fitting methods, such as bilinear interpolation and nearest neighbor interpolation, were the first to address the issue of lower spatial resolution caused by the larger sensor spatial distance (sparse distribution) [49].

For example, researchers in [38] proposed a regression model to reconstruct a high-resolution plantar pressure distribution map using less than nine sensors. The model, named the sparse-sensing continuous plantar pressure model (SCPM), employed a Gaussian mixture model (GWM) as the basic function. The model was trained using high-resolution plantar pressure data in the principal component analysis (PCA) method. The obtained model utilized linear least squares (LSQ) to calculate free parameters and reconstruct the pressure distribution. In the experiment, when the number of sensors exceeded 7, the number of GWM mixtures was set to 40, and the dimensions of the vector in GWM were set to 10. Under these conditions, the root mean square error (RMSE) could be kept below 20 kPa. Furthermore, the RMSE gradually decreased with increasing sensor spatial distance.

Another example is provided by researchers in [50]. They developed a novel sitting mat with 567 PSD sensing arrays made from E-textile. In the data processing stage, they interpolated nine data points between the raw data, thus enhancing the virtual resolution up to 43,376. This increased resolution greatly contributed to the mapping of pressure distribution.

Aside from spatial fitting, temporal fitting is another method. In the insole system developed by researchers in [39], they initially divided the insole into six regions and then obtained the complete pressure distribution by superimposing these regions. In the data processing stage, the resolution is improved by interpolating plantar pressure data based on the temporally adjacent average plantar pressure. The interpolated data is further filtered using median filtering. This method facilitates the mapping and calculation of peak pressure, pressure-time integral, and center of pressure.

To adhere to the Nyquist sampling theory, the spatial distance between sensors and the number of sensors must maintain a minimum value and cannot be further reduced. Meanwhile, the fitting method considers the plantar pressure distribution of the entire area as the superposition of individual regions, such as the heel and toes. Although this method simplifies the calculation, neglecting physiological differences caused by this approach can compromise the accuracy of reconstruction [37,39].

### 3.2. Compressive Sensing

To tackle the aforementioned issues, compressive sensing (CS) has been employed in gait monitoring based on the following theory: by studying the features of PSD, researchers discovered that in domains where mapping is sparse, PSD can be represented by combining a group of specific linear models [41].

In [41], researchers introduced a compressive sensing technique for reconstructing PSD. Firstly, they separated the steps of the participants and aligned the pressure images of those steps. Secondly, they employed high-resolution image data to train models using supervised dictionary learning. The dictionary was designed to transform high-resolution PSD into a sparse representation, which is a combination of several linear models. The obtained results were then used to reconstruct PSD in two ways: orthogonal matching pursuit (OMP) or least absolute shrinkage and selection operator (LASSO). Their results demonstrate that the CS reconstruction method yields accuracy comparable to that achieved by using interpolation with 170 sensors, using only data from 4 sensors. To be precise, when utilizing 4 sensors, the root mean square error (RMSE) is 6.7 kPa per sensing unit. This error can be kept below 7 kPa when the external pressure is below 160 kPa. Additionally, when the number of sensors is less than 9, employing larger sensors can result in a lower RMS error (7 kPa) compared to using smaller sensors (9 kPa).

Another example of CS reconstruction is provided in [42]. Researchers initially collected plantar pressure images (PPI) from force plates during normal walking speed to obtain sparse representations of PSD. Then, they utilized the Topelitz measurement matrix as the CS classifier to categorize the PPIs. The achieved classification accuracy was 97.76%.

### 3.3. Machine Learning

The compressive sensing and fitting techniques can accurately reconstruct PSD in many cases, but their common drawback is the lack of interpretability in the reconstruction. To extend the application of PSD to a wider range of medical scenarios, it is crucial to emphasize the correlation between plantar pressure and clinical symptoms [51,52]. As a result, various machine learning methods have been utilized to improve performance in analyzing more complex and time-varying spatial gait parameters.

For this purpose, researchers in [45] developed a portable, stretchable, and flexible insole monitoring system based on an active-matrix sensing array (AMSA). This array is constructed on a PCB and utilizes PVDF as a sensing material, ensuring its stability and ability to detect shear pressure [9]. To provide more detailed medical data, they employed a support vector machine (SVM) model to classify five common human postures and actions: jump, walk, jog, squat, and half squat [45]. In the process, they initially considered the average output voltage as the eigenvalue of the five motions due to their distinct differences. They then selected 38 samples from a total of 45 samples for each motion as the training dataset, while the remaining 7 samples were used for prediction. The results revealed a classification accuracy of 99.2%. Furthermore, when classifying 62 clinical samples of patients with Lumbar degenerative disease, the accuracy reached 100%, highlighting its potential application in the diagnosis of chronic diseases.

## 4. The Feature Extraction of Plantar Pressure from Gait Data

The reconstruction of plantar pressure provides abundant data on the gait cycle. Diagnosis-based features, such as peak plantar pressure and stance phase length, require feature extraction. Therefore, extracting gait features is a crucial step in gait data processing. In the early stages, researchers focused on quantifiable gait features. For example, Barton et al. [53] obtained 1316 plantar pressure parameters using force plates, while Wolf et al. [54] measured 3670 types of gait features from a single gait cycle. Years later, Begg et al. proposed several principles for feature extraction [36]. Firstly, desired features should be observable, such as the maximum and minimum pressure in one gait cycle. Secondly, the desired data should be general, meaning these features occur in all types of participants. This second principle is particularly important for machine learning methods, as the generalizability is directly related to the robustness of the machine learning techniques [36]. In recent years, machine learning-based techniques have been employed for feature extraction [37].

Generally, based on the aforementioned reasons [36,37], techniques for extracting gait features can be classified into three categories: Fourier Transform (FT), Direct Calculation (DC), and Machine Learning (ML). Table 2 [55,56,57,58,59,60,61,62,63,64,65,66,67] provides a brief overview of the examples in Section 4.

### 4.1. Fourier Transform

FT refers to the process of transforming the reconstructed plantar force distribution map into a digital image format. The image is then manipulated in the frequency domain before being transformed back into the spatial domain [68]. An illustrative example is presented by researchers in [69]. Their study concentrates on diabetic foot and insole temperature. Since diabetic foot and ulceration can significantly affect insole temperature, specifically when the insole temperature rises by more than 2.2 °C, it can be considered a symptom of diabetic foot. Therefore, temperature can be viewed as a reliable feature in this context.

Initially, they utilized RGB channels to create thermal maps of insole temperature. They then performed a Fourier Transform (FT) on the digital images and generated a random noise image of the same size as the amplitude matrix obtained from the FT. The amplification range of the noise image is uniformly distributed and varies from 0 to 1. These generated images exhibit noticeable differences in insole temperature. Finally, the inversely transformed images were included in both the training dataset and the test dataset. The results demonstrated that by employing three models (ResNet, DFT-Net, Transformer), the classification accuracy can achieve 100%, 97.06%, and 88.24%, respectively.

The FT can also be used to select and filter features that are highly correlated with chronic diseases. Freezing of gait (FOG) is the most typical clinical symptom of PD, characterized by the inability to move the foot during walking, as if the lower limbs were frozen [55]. Therefore, the diagnosis of FOG is closely related to plantar pressure [70]. Hence, selecting suitable gait features is crucial for accurate diagnosis. Currently, the most commonly used feature is the freeze index (FI), which employs FT to quantify the extent of leg freezing [56].

In [57], researchers utilized Wavelet Transform (WT, *n* = 14) and FFT (*n* = 8) to calculate features, where n represents the length of the sampling window. Following the computation, a total of 31,839 windows yielded 457 features. To select the desired features, the minimum-redundancy maximum-relevance (mRMR) filter was applied. This algorithm identifies the inputs that are most closely related to the class representing FOG in a multivariate manner. Their results demonstrated that FT, FFT total power, and mean detail coefficient are the top three features correlated with FOG.

### 4.2. Direct Calculation

Aside from FT, calculating features directly from gait data is another effective method for analyzing gait parameters [37]. The DC means can be obtained through various techniques, including peak detection [58,59], threshold division [60,61], weighted average [62,63], and summarization [65,66].

Peak detection aims to capture the extreme maximum or minimum of the output voltage signal. In 2009, Waaijman et al. [58] utilized PSD sensor data to calculate peak pressure and achieved an RMSE accuracy lower than 2.5 kPa. In an effort to enhance the medical information obtained from gait features, Potluri et al. [59] divided the insole into eight regions based on anatomical knowledge. They further employed 3-D insole graphic visualization to improve the distribution images of plantar pressure. To capture the peak pressure, cadence time, and stance ratio, they utilized an LSTM model. Their algorithm successfully differentiated between normal participants, spastic participants, Parkinson participants, and ataxic participants based on four gait features (stance ratio, step time, cadence, and peak pressure).

(1) $$COP_{x}=\frac{\sum_{1}^{64}F_{i}X_{i}}{VGRF}\;COP_{y}=\frac{\sum_{1}^{64}F_{i}Y_{i}}{VGRF}$$

Threshold division is an approach in digital image processing that determines a grayscale threshold to divide the image into black and white regions. This method can be applied to calculate temporal gait parameters. For example, in [60], researchers calculated the area of plantar contact with the ground using threshold segmentation. They then derived the temporal parameters of gait from the duration of these areas, achieving a low level of error. The RMSEs for stride time, swing time, and velocity were 0.017, 0.019, and 1.74, respectively. Furthermore, thresholds can also be used to calculate spatial features. In [61], researchers first established the force of each sensing point based on the output voltage threshold value (−0.02 V). They then calculated the vertical ground reaction force (VGRF) as the superposition of forces at each sensing point. Additionally, they utilized the following equation to calculate the center of pressure (COP). Fi represents the force of each sensing point, and X and Y represent the distance in the forward and lateral directions, respectively.

The weighted average method is particularly suitable for calculating the COP as it establishes a relationship between lateral or anterior distances and force or plantar pressure, as shown in Equation (1) [61,62]. In 1997, Besser et al. employed the weighted average method to calculate the COP, resulting in an RMSE lower than 13.8 mm [62]. In 2019, Weizman et al. developed an insole system with 88 piezoresistive ink-based sensors [63]. They first depicted the force graph over time and then calculated the gradient in the X (lateral and medial) and Y (anterior and posterior) directions to determine the weights. They compared their COP values with those obtained using a commercial insole system, Pedar-X. Their results showed an RMSE under 4mm in the X direction and under 10mm in the Y direction.

Summarization can be applied to the calculation of Ground Reaction Force (GRF) [64,65]. It focuses on the superposition of values gathered from sensing points. In 1998, Davis et al. [64] utilized an insole system with 16 sensors distributed in a 4 × 4 matrix. In this case, a simple summarization yielded accurate results. The relative error of linearity was 5%, and the hysteresis was controlled below 7.5%. Furthermore, the difference between experimental results and ground truth results was only 5%. As sensing techniques improved, the accuracy also increased. In 2012, Lucas et al. developed a three-axis GRF measuring insole system [65]. They employed silicone as the sensing material as pressure can be reflected through the intensity of light, thereby affecting the current value from the semiconductor due to the photoconductive phenomenon. The output GRF was the linear combination of the responses of opto-electronic detectors, as shown in Equation (2). The mean error was less than 10.7 N when the shear pressure was 68.7 N.

(2) $$Ground\;Reaction\;Force=\sum \alpha_{i}D_{i}E_{i}$$

In the equation above, $\alpha_{i}$ represents the coefficient calculated from the input signal, while the $D_{i}\;E_{i}$ refers to the response from the detector I and photoemitter I, respectively.

### 4.3. Machine Learning

Though direct means can effectively calculate many parameters, the non-linearity in those algorithms still hinders the accuracy of parameter estimation [37]. Therefore, some machine learning-based indirect means can be applied. For instance, the rotation angle of joints in the lower limbs can reflect the risk of injury, which commonly occurs in workplace and daily living scenarios, necessitating injury prediction.

To address this, Zachary et al. proposed an insole system with six force-sensing resistors [66]. They implemented a Gaussian Process Regression (GPR) linear regression as their model, collecting a finite number of time variables, with each sample of this time series containing α number of features. Their results effectively predicted the hip angle, knee angle, ankle angle, and lumbosacral joint angle (L5S1). Among these angles, L5S1 exhibited the lowest RMSE, with the X-axis and Y-axis having RMSE values of 0.21° and 0.22°, respectively.

By changing the model and algorithms, the applications for the data can also be modified. Initially, Anderson and Zachary et al. utilized a Principal Component Analysis (PCA) to simplify the input data to a manageable level (18 original features) [67]. Then, the data was processed using TensorFlow and K-Nearest Neighbors (KNN) algorithms. The researchers believe that this combination can yield the most homogeneous classification since their algorithm is trained to choose distinct values of K and select the nearest neighbor, ensuring correlation. Ultimately, their model can predict and classify motions such as walking, descending, running, and falling (back, front, left, right) with an average accuracy of 86%.

### 4.4. Comparison and Choice Explanation of Gait Feature Extraction Methods

The methods mentioned above provide ample examples about extracting gait features from gait parameters. This subsection aims to summarize and discuss these methods.

The Fourier transform can extract peak pressure and spatial parameters like freezing index (FI) because the DFT can reconstruct time-domain and spatial-domain signals from the digital signal of the sensor [55,56,57,70].

The branches of the direct calculation method, including threshold segmentation, weighted averaging, and summarization, are advantageous due to their computational convenience. These algorithms can quickly compute peak pressure through threshold segmentation and calculate a distribution map of stress, shear stress, normal stress, and the center of pressure (COP) rapidly, by utilizing weighted averaging and summarization techniques [60,61,62,63,64,65].

Machine learning algorithms, such as LSTM and Transformer, can handle signals in time-series data and make predictions and classifications on sequential information. Therefore, machine learning algorithms can classify the motion status of users of insole force sensors, predict temporal parameters such as the ankle angle as it changes over time and insole temperature [55,66,67].

## 5. The Normalization of Gait Data Parameters

Based on the aforementioned procedures, the reconstruction of plantar stress and the extraction of gait features can be achieved. However, gait features are the result of the collective action of muscles, bones, lower limbs, and nerves, meaning that the parameters in gait characteristics are closely related to personal body parameters, such as weight and leg length [37]. Therefore, normalizing gait data to reduce individual differences is necessary [36]. Additionally, when applying machine learning methods in diagnosis, it is important for the feature values to be confined to a certain interval, e.g., [0, 1]. Hence, according to [36,71], algorithms for gait data normalization can be classified into two branches: data transformation and anthropometric scaling [36]. Table 3 provides a brief overview of the examples in Section 5.

### 5.1. Data Transformation

First of all, data transformation usually maps data with a large range of variation (such as peak plantar pressure) to a fixed interval (such as [0, 1], or [–1, 1]). Such transformations are frequently performed in neural networks [54]. Two of the most commonly used algorithms are: minimum-maximum normalization (MMN) and zero-mean normalization (ZMN).

(3) $$x^{\prime }=\frac{\left(x-x_{min}\right)\times \left(L_{max}-L_{min}\right)}{\left(x_{max}-x_{min}\right)}+L_{min}$$

(4) $$x^{\prime }=\frac{x-\mu }{\sigma }$$

Equation (3) describes the MMN, and Equation (4) describes the ZMN. In the expression of MMN, x represents a certain gait parameter, and the subscripts min and max represent the minimum and maximum values in the x dataset, respectively. The minimum and maximum values of L represent the desired data interval, such as [0, 1]. In many neural network algorithms, the input and output data need to be converted between [0, 1]. Therefore, this method can enhance the accuracy of applying machine learning in gait data analysis [53,72]. For instance, this transformation can be used to normalize the step length or stride length of individuals with varying lengths. Typically, the stride time represents the time interval between two consecutive heel strikes on the ground. Therefore, researchers set Lmax as the gait cycle time and Lmin as 0, and the x dataset is generated from the stride time. Consequently, the obtained x’ dataset represents the proportion of stride time (or swing phase) in the entire gait cycle time, effectively reducing individual differences [73,74].

In the ZMN expression, the dataset x is transformed into x’ with a mean of 0 and a variance of 1, where μ represents the average value of the x dataset, and σ represents the standard deviation of the x dataset. Although the original dataset does not necessarily follow a normal distribution, this calculation method is suitable when comparing the data of one patient with the data of other patients or with the data of a large population [36]. For example, researchers used this transformation and PCA to formulate gait parameters in a study involving patients who had suffered from a stroke [75]. In the experiment, the researchers collected 40 variables from each patient, including stride speed, stride length, walking speed, mechanical energy per cycle, etc. After normalization, all variables had the same range [0, 1], and the subsequent PCA identified speed as the first principal component with a variance ratio of 40.8%.

### 5.2. Anthropometric Scaling

Normalization can restrict data to a certain interval. However, intrinsic anthropometric features can also introduce variability within gait parameters [36,76]. For instance, individuals with a higher body weight generally exhibit greater peak plantar pressure, ground reaction force, and joint forces, while the center of pressure can also differ. Therefore, the pressure threshold method alone cannot be used to diagnose potential patients with diabetes [71]. Additionally, individuals with longer legs tend to have higher gait velocity and lower cadence [77]. Hence, when using gait speed as a diagnostic factor for PD, leg length should be taken into consideration [78].

To address these issues, anthropometric scaling can be employed. The concept behind this scaling normalization is to transform relevant gait parameters using functional expressions. The arguments of the function encompass both anthropological parameters, such as weight and leg length, and measured parameters that share the same dimension, such as shear stress and normal stress [76].

So far, the most widely used transformation functions were proposed by Hof in 1996 [76]. In this work, key gait parameters were converted into non-dimensional quantities. Physical quantities such as mass and length were transformed into dimensionless measures using a simple ratio method. For more complex physical quantities like acceleration, velocity, force, and energy, functions were formulated incorporating parameters such as leg length, mass, and acceleration due to gravity. The main parameters and their corresponding explanations are provided in Table 3.

**Table 3:** Main parameters in gait data scaling [76].

Parameters	Quantity Symbol	Scaling Equation	Additional Explanation
mass	$m\;and\;m_{0}$	$m^{\prime }=\frac{m}{m_{0}}$	m represents the value with mass quantity, including mass. *m*_0_ is the body mass
length	$l\;and\;l_{0}$	$l^{\prime }=\frac{l}{l_{0}}$	l represents the value with length quantity, including stride length l0 represents the leg length
time	T	$t^{\prime }=\frac{t}{\sqrt{g/l_{0}}}$	Most of the time studied in gait is related to velocity, which is affected by leg length
speed	V	$v^{\prime }=\frac{v}{\sqrt{gl_{0}}}$	Legs of people have different length; the length can influence the velocity
acceleration	a	$a^{\prime }=\frac{a}{g}$	Most of the accelerations studied in gait are related to gravity
force	F	$F^{\prime }=\frac{F}{m_{0}g}$	Force is divided by body weight and the direction of force can be changed with gravitation
energy	W	$W^{\prime }=\frac{W}{m_{0}gl_{0}}$	Work is defined as the product of force and length
angle	∅	∅	∅ is dimensionless

### 5.3. Comparison and Summarization of Data Transformation and Anthropometric Scaling

Section 5 provided two branches of gait parameters normalization: data transformation and anthropometric scaling. This subsection aims to present a brief summarization of their application scenarios and mechanisms.

The data transformation utilizes normalizing equations like ZMN and MMN (Equation (3)). This method possesses simplicity in computation, allowing for the normalization of all types of gait parameters and their application to datasets for machine learning algorithms [72,73,74].

However, the normalization of ZMN and MMN is only based on the dataset obtained from the sensors and does not take into account the influence of individual body parameters on gait. For instance, individuals with different leg lengths may have different gait speeds. Therefore, when using machine learning algorithms to investigate gait speed for assisting in Parkinson’s disease diagnosis and treatment, factors such as leg length should be considered [71,77,78].

As a result, anthropometric scaling has been proposed, which comprehensively introduces normalization formulas for parameters such as mass, force, velocity, time, and angles while considering individual body parameters [76]. Table 3 presents those equations. Thus, the processed data take into account individual differences, allowing for better assistance in medical diagnosis and treatment.

## 6. Six Chronic Diseases and Corresponding Insole Systems

Based on the aforementioned techniques, numerous insole systems have been developed, providing precise data on plantar pressure and humidity. These data contribute to the calculation of temporal and spatial gait parameters such as gait frequency, gait velocity, step length, and duration phase, among others. These parameters play a crucial role in rehabilitation and diagnosis for patients with various diseases.

**Figure 5:**
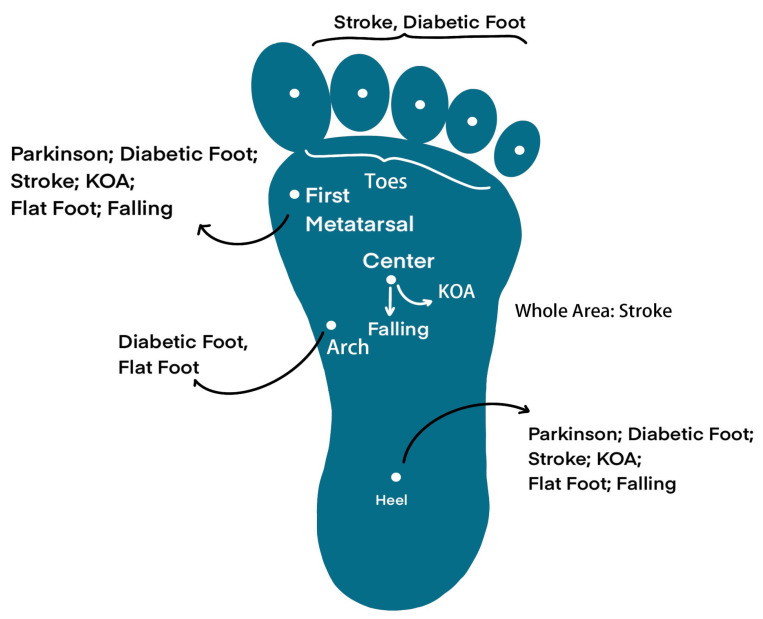
Desired sensing points on the foot, they are suitable for different diseases.

**Figure 6:**
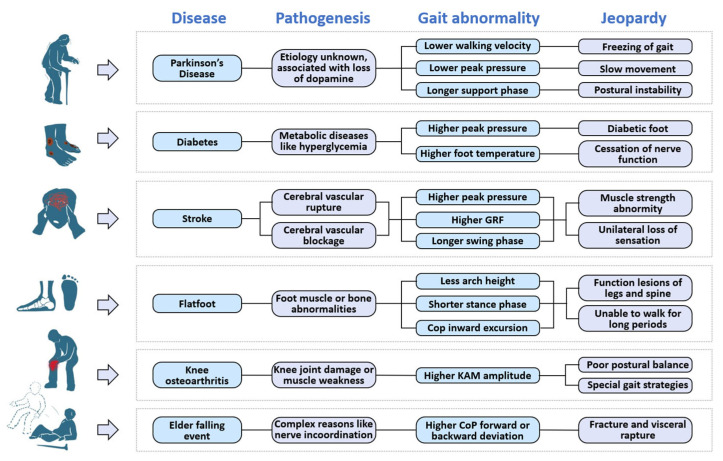
Demonstrations of six chronic diseases mentioned. Each disease has different pathogenesis and causes different gait abnormaility with correspondent jeopardies.

In this section, we will introduce two types of neurological diseases (PD and stroke), two types of orthopedic diseases (knee osteoarthritis and flat foot), and two types of musculoskeletal diseases (diabetes and elderly falling), along with their corresponding insole systems. Furthermore, an illustration of gait features is presented in Figure 4. Figure 5 provides a depiction of the sensing points for each disease, and Figure 6 showcases pathogenic demonstrations of chronic diseases.

### 6.1. Explanation of Gait Features

Insole systems acquire a wide range of gait parameters through detection and measurement, which serve as the basis for gait feature analysis. However, the definition of gait features is not universally specific. Therefore, this subsection is dedicated to explaining four key gait features: peak plantar pressure, center of pressure (CoP), gait phases, and length of steps and strides. It is worth mentioning that these features (excluding step length) are typically measured as a single value on both sides of the sagittal plane, considering the symmetry of gait.

According to its definition, as depicted in Figure 4, peak plantar pressure refers to the maximum pressure exerted by the sole of the foot on the ground. Abnormal peak pressure is considered a potential indicator of various chronic diseases, such as Parkinson’s disease and diabetic foot [79,80].

The swing and stance phases refer to the duration of time that a foot spends in the swinging motion and the time it remains in contact with the ground, respectively [76,77].

Stride length represents the distance between two consecutive foot contacts of the same foot, while step length represents the distance between the points where the two feet make contact with the ground [80,81].

Among insole systems for flatfoot, KOA, and falling, the CoP is commonly measured. However, it should be noted that the interpretation of CoP can vary depending on the specific scenario, and thus requires clarification.

CoP originally refers to the point where the ground supports the sole of the foot (as shown in Figure 4). CoP can also represent the center of plantar pressure due to Newton’s third law (interaction forces are equal in magnitude and opposite in direction) [82,83].

### 6.2. Parkinson

PD is a common neurodegenerative disease among middle-aged and elderly individuals [84]. It is characterized by the gradual degradation of dopamine-producing cells, leading to disruptions in muscle movement control and resulting in symptoms such as freezing of gait (FoG), slow movement, and postural instability [85]. Medical treatments for PD typically involve dopamine replacement therapy or surgery, but postoperative responses can vary significantly among individuals and may exhibit unpredictable fluctuations [86]. Therefore, there is a critical need for effective postoperative monitoring and treatment methods for PD patients. In previous studies, various wearable devices based on gait analysis have been developed to address this need. These devices focus on parameters such as gait velocity, stride length, duration of gait phases, and plantar pressure distribution. Table 4 provides typical values for these parameters in PD patients and healthy individuals [87,88,89].

The research of wearable insole systems for PD is divided into two parts: parameters monitoring and external rhythmical cues stimulation therapy [90]; each part will be discussed in this section.

Sensors for measuring parameters in PD can be classified into two categories: inertial sensors (such as gyrosCoPes and accelerometers) [91,92] and plantar pressure sensors [93,94,95,96]. In the case of inertial sensors, R. Hua et al. [91] proposed a PD monitoring insole that incorporates two accelerometers positioned at the heel and the first metatarsal of the foot. The vertical component of the measured acceleration is utilized to detect gait events and determine phase durations, while the horizontal component is used to calculate gait velocity and stride length. The system achieved an average detection accuracy of 88.74%. Additionally, the insole system can differentiate between the “activity state” and “inactivity state” of patients, allowing for the adaptation of the system’s working mode to conserve battery power. B. Jens et al. [92] attached an inertial measurement unit (IMU) sensor to the lateral heel area outside a shoe to assess the degree of gait impairment in PD patients. Gait features in both the time and frequency domains are extracted, and a linear discriminant analysis (LDA) model is employed for classification. The results indicated that the gait cycle, movement range, and signal energy in the frequency domain (0.5–3 Hz) are the most suitable indicators of PD-related gait impairments.

The use of plantar pressure sensors is also applicable in PD research. K. Grandez et al. [93] developed an insole system equipped with three force-sensing resistors (FSRs) distributed at the forefoot and heel areas to compare gait parameters between PD patients and healthy individuals. The captured signals are visualized through a PC application and subsequently analyzed by medical professionals. The results indicated that PD patients exhibit a higher force central frequency (around 1.8 Hz) compared to healthy individuals (around 0.8 Hz), and the plantar peak pressure amplitude of PD patients is approximately 40% lower.

As for adjuvant therapy, wearable devices are employed to provide external stimulation cues to PD patients. These cues can be categorized into three main types: visual, auditory, and somatosensory. Visual cues often utilize laser-assisted devices (LADs). For example, E. Lim et al. [97] used a handheld LAD as a walking aid for PD patients. When patients experience freezing of gait or hesitation, the LAD projects a meter-wide red line ahead to provide instructions. After two days of training, the gait cycles of three patients were experimentally reduced from approximately 10 s per step to less than 5 s per step. Delivering auditory stimulation is another option for PD treatment.

In a previous study by E. Jovanov et al. [98], a therapy system was developed consisting of a stimulation headset and an on-shoe inertial sensor. The inertial sensor detects freezing of gait (FoG) events in real-time by analyzing the signal energy distribution pattern in the frequency domain. The stimulation headset then plays rhythmic auditory recordings, such as a ‘click’ sound, to unfreeze the patients’ gait. The system achieves effective therapy with a latency of 580 ms.

Similarly, Marc Bachlin et al. [99] utilized an earphone and three on-body acceleration sensors placed at the ankle, thigh, and waist to detect and treat FoG. The final detection sensitivity reached 73.1% in 237 tests. While visual and auditory external methods enhance therapy and diagnosis effectiveness, they both have limitations in certain environments. Acoustic cues, like music beats or metronomes, can be disrupted by surrounding noise, while visual-based methods heavily rely on natural lighting conditions [100].

To address these limitations, somatosensory rhythmic external cue-based systems have been proposed. For example, a previous study [101] demonstrated a step-synchronized vibration system with three vibratory devices fixed at high-pressure insole areas (heel and metatarsus). The vibratory motors are activated when the foot is in contact with the ground. The results showed improvements in patients’ gait cycle, step length, and gait velocity, with a coefficient of variation (CV) of approximately 0.05.

The mentioned research not only provides substantial evidence for diagnosis but also enhances surgical rehabilitation, contributing to the treatment of PD.

### 6.3. Diabetes

Diabetes is a group of metabolic diseases characterized by hyperglycemia. With the improvement of living standards, the incidence of diabetes has been continuously increasing over the past few decades. In 2005, the International Diabetes Federation warned that by 2030, one in ten people would have diabetes [102]. Diabetes can lead to various complications, with diabetic foot being the most severe. If not promptly treated, diabetic foot can progress to a life-threatening condition that may require amputation [103]. Diabetic foot is characterized by two notable pathological symptoms: inflammation and dryness. Inflammation indicates ulceration of the tissues in the affected area, while dryness indicates the loss of autonomic nervous function [104,105].

Early-stage research on diabetic foot focused on the detection of foot ulcers. In 1986, M. E. Edmonds et al. [106] observed that diabetic foot ulcers occur more frequently at the forefoot and toes compared to other areas of the sole. In 1998, D. G. Armstrong et al. [107] established a strong correlation between high plantar pressure (≥60 kPa) and ulceration in individuals with diabetes. A recent study [108] demonstrated that patients with foot ulcers exhibit a rolling motion of the foot during the mid-stance phase of walking. Building upon these medical findings, insole systems for plantar pressure measurement have been developed for monitoring foot ulcers. For example, P. Aqueveque et al. [109] developed an insole system that measures pressure at high-stress areas. It incorporates eight capacitive sensors with diameters of 15 mm strategically placed at the toes, metatarsus, arch, lateral midfoot, and heel. Each sensor unit is capable of withstanding weights up to 20 kg. In another design [110], a micro-electronic mechanical smart portable system (MEMS) based on piezoresistive techniques was proposed for the detection of foot ulcers. The sensor achieved a wide sensing range of 0–2 MPa.

Moreover, recent studies have aimed to investigate the impact of individual patient differences on the pressure at ulcerated areas. M. J. Hessert et al. [111] have highlighted that younger individuals and older individuals exhibit different plantar pressure patterns. Younger individuals tend to experience lower pressure on the lateral area of the foot, which may contribute to better body stability during walking. M. Nouman et al. [112] utilized the Pedar-X^®^ in-shoe pressure measurement system (based on capacitors) to analyze the relationship between plantar pressure distribution patterns and patients’ weight. The findings revealed that the midfoot peak pressure is significantly higher in the obese group compared to other patients. Therefore, for a more accurate diagnosis of ulceration, additional physical factors of patients should be taken into consideration.

Apart from plantar pressure amplitude, foot temperature and humidity also play a significant role in diabetic foot detection as they are closely associated with foot dryness. T. Bernard et al. [32] developed an insole sensor system for the simultaneous monitoring of pressure and temperature in diabetic feet. The system included three thermistors and three pressure sensors placed at the big toe, first metatarsal, and heel. Comparative experiments between diabetic patients and healthy individuals revealed that a plantar pressure above 60 kPa and a temperature difference exceeding 2.2 °C (between different sensor locations) were indicative of early foot ulcers. M. Anouar et al. [113] designed a flexible insole with three integrated temperature/humidity SHT1X sensors and eight inductive force sensors. The collected data, including plantar pressure, humidity, and temperature, are displayed to patients and doctors through a mobile phone application. Real-time alerts on the severity level of patients’ foot ulcers are provided using threshold-based methods.

### 6.4. Post-Stroke Rehabilitation

Stroke, an acute disease caused by the rupture or blockage of cerebral blood vessels, is a leading cause of disabilities among elderly individuals worldwide [114,115]. Approximately 80% of post-stroke patients experience various degrees of motor impairments, including abnormalities in muscle strength and sensory loss in unilateral limbs [116]. To aid in the recovery of motor functions and enhance the quality of life for these patients, there have been ongoing efforts to develop wearable kinematic assessment systems that monitor daily activities and assist in formulating clinical treatment plans.

The main focus is on the symmetry of limb actions during walking. Typically, the affected side of the lower extremity in stroke patients exhibits relatively lower plantar pressures, resulting in longer stance phases, longer support phases, and lower plantar pressure amplitudes. These parameters are summarized in Table 5 [117,118].

Various limb action monitoring systems have been reported for post-stroke rehabilitation. For example, P. L. Meyer et al. [119] developed a shoe-based wearable system with five FSR sensors (located at the toes, metatarsus, and heel) to capture gait phases in stroke-impaired individuals. The sum of the detected pressure is used to identify heel-strike and toe-off events, enabling the calculation of phase durations. Through experiments involving 16 healthy subjects and 7 post-stroke subjects, the system demonstrated a 95% confidence level in gait phase detection.

In terms of measuring shear pressure, piezoelectric sensors can be utilized. A. M. Howell et al. [120] developed a 32-sensor insole to measure the ground reaction force and ankle moment in stroke patients. The ground reaction force was calculated by summing the forces from each sensor, while the ankle moment was determined by multiplying the force of each sensor by its anterior-posterior distance to the ankle joint center. Results indicated that patients with impaired gait exhibited distinct patterns in terms of energy concentration and peak numbers in the signals.

Moreover, to ensure optimal fit and comfort for patients undergoing rehabilitation, L. Qin et al. [121] introduced innovations in the design of insoles by developing 3D tailor-made insoles. The patients’ feet were scanned to create 3D models using specialized software. The insole structures were then simulated and manufactured through silicone solution casting. Experimental results obtained from patients demonstrated that the peak pressure measured in the mid-foot area was higher compared to traditional flat insoles, indicating improved contact between the insole and the foot. This customization approach enables a more precise and personalized rehabilitation for post-stroke patients.

M. Organero et al. [122] conducted research on optimizing the quantity and placement of sensors, an often overlooked aspect in this field. Initially, an insole with eight FSR sensors was used as the baseline configuration. However, they achieved a more cost-effective solution by minimizing the L1 distance of plantar pressure patterns measured by all eight sensors. The revised system utilized a combination of four sensors, specifically positioned at locations 1, 4, 6, and 7. This reduction in the number of sensors resulted in a 50% cost reduction, which is significant for both stroke survivors and commercial companies. In addition to sensor layout, there are other aspects that warrant optimization. For instance, in hardware design, the selection of economical and practical microchips has not yet yielded satisfactory results and requires further research.

### 6.5. Flatfoot

The arch is an essential structure of the human foot as it provides stability, absorbs impact forces, and facilitates the support and propulsion of the body [123]. Flatfoot is a common foot deformity characterized by the collapse of the arch, which can be attributed to abnormalities in the foot muscles or bones. This condition hinders patients from engaging in prolonged walking and other lower limb exercises, and if left untreated, it can lead to functional impairments in the leg and spine [124]. Consequently, early detection of flatfoot is crucial for initiating effective rehabilitation interventions.

The clinical diagnosis of flatfoot relies on two types of information, as outlined in Table 6 [125,126,127,128]. The first category includes arch structure parameters, such as height, width, and the contact area between the plantar surface and the ground [127]. The second category involves gait features such as gait phases, center of pressure (CoP), and rotation angles of the ankle joints [129]. Previous studies have employed various methods and techniques to measure these parameters.

The conventional techniques primarily focus on measuring the plantar structure. For instance, in [125], an ultrasonic distance sensor was utilized to scan the entire arch area and determine the maximum distance between the arch and the ground. Patients with an arch distance of less than 1 cm were diagnosed with flatfoot. The results aligned with the assessment of a specialized physician among 20 subjects. A. O. Hamza et al. [126] proposed an optical footprint photography method and related algorithms to provide a more specific evaluation of flatfoot. The patient’s foot surface was placed on a transparent panel and photographed from the opposite side using a camera. The captured image was converted into binary form and divided into plantar contact and non-contact areas. The degree of foot deformity could be diagnosed and classified into seven levels based on the ratio between these areas. In his work [130], Luis A. Navarro developed a flatfoot detection platform consisting of a webcam and six FSR sensors, aiming to provide comprehensive information about the foot structure. The webcam collected data on arch length and width, while the force sensors measured the pressure at discrete plantar regions. By combining these two types of information, the pressure distribution pattern across the entire plantar surface could be estimated. The output results of this system were provided to specialists as an analysis tool.

Although several effective diagnostic platforms have been proposed, these techniques are often associated with complex data collection processes and large volumes of data. To address this issue, J. Y. Kim et al. [131] introduced an in-shoe sensing technology. The new system incorporates two force sensors and one angle sensor based on piezoresistive techniques to measure gait-related signals. The front and rear force sensors detect the toe and heel strike or lift-off events to identify different gait phases, while the angle sensor provides information about ankle rotation angles. Using a deep neural network model, the presence of flatfoot could be determined with an accuracy of 81.52%. The in-shoe force-sensing method offers advantages in terms of comfort and convenience; however, its accuracy is limited since it only measures gait-related features. In the future, it is anticipated that portable flatfoot diagnostic equipment will be developed, integrating the measurement of both foot structure and gait features.

### 6.6. Knee Osteoarthritis

Knee osteoarthritis (KOA) is a common chronic disease characterized by knee joint damage and muscle weakness [132]. Patients with KOA often exhibit poor postural balance and adopt specific gait strategies. Several critical indicators are used to monitor and analyze the motion abnormalities in KOA patients, including plantar pressure distribution [133], center of pressure (CoP) path [134], duration of gait phases, and knee adduction moment (KAM) [135,136]. These indicators are summarized in Table 7 [134,137,138]. KOA patients typically exhibit a more dispersed plantar pressure distribution pattern, longer duration of the single support phase, smaller range of CoP path, and higher KAM values during walking.

On the basis of medical studies, insoles with force-sensitive sensors have been designed for KOA patients. For example, I. Saito et al. [134] utilized the F-scan insole sensing system to detect the CoP path in KOA patients. In walking experiments, it was observed that the percentage of the anteroposterior CoP path length to the foot length (%Long) was significantly lower in KOA patients compared to normal individuals (52.4% vs. 64.7%). This difference can be attributed to the limited knee movement angles in KOA patients, resulting in a shorter anteroposterior displacement of the CoP.

M. M. Organero et al. [137] employed an insole system with eight force-sensitive resistor (FSR) sensors to analyze the walking strategy of KOA patients. Through experiments involving 14 KOA patients and 14 healthy individuals as the control group, it was found that the duration of the double support phase and the transfer mode of forefoot pressure (e.g., movement speed and route) are closely related to KOA. Each of these features was able to identify KOA with an accuracy higher than 89%, using support vector machines (SVM) and decision tree algorithms.

In addition to plantar pressure and CoP path, KAM has also been proven to be a critical indicator of KOA. S.H. Kang et al. [138] developed a system consisting of a six-axis goniometer and a six-axis force/torque sensor to measure the KAM of both KOA patients and healthy individuals during lower limb exercises. In the experiment, which involved stepping on an elliptical trainer, it was found that the mean peak KAM value of the patient group was 47% higher compared to the healthy group.

Another study [139] proposed a wearable feedback system to reduce the KAM value of KOA patients during walking. The system comprised an insole FSR sensor located at the lateral side of the heel and a vibration motor at the ankle. When the detected pressure crossed a pre-determined threshold, the motor was activated to assist patients in adopting a more medial weight-bearing strategy. The results demonstrated that through this feedback mechanism, reductions of 6.0% and 13.9% were achieved in the first and second peak of the KAM, respectively.

In a study by A. M. Howell et al. [140], an insole with 12 FSRs was designed to estimate patients’ KAM. The obtained plantar pressure data was compared with KAM values simultaneously measured by motion capture cameras. Using a linear regression method with a correlation index of 0.80, this KAM detection method achieved an accuracy of over 75%.

In the future, there is a desire for insole systems to provide accurate measurements of both plantar pressure and KAM. This would enable a more comprehensive and reliable diagnosis for KOA patients.

### 6.7. Elder Falling Event

The prediction or warning of falling events, which can result in life-threatening injuries for elderly individuals such as fractures and visceral ruptures, is highly desired in elderly support services [141,142,143,144]. Gait information, including CoP displacement, total CoP displacement, step length, and gait velocity, is considered important for detecting falls [145,146]. These parameters are explained in Table 8 and Section 6.1. Among those parameters, ground reaction force deviation and CoP deviation (observed through gait phase and duration) are often measured for fall detection [145,146].

Early-stage studies involved observing the gait of elderly individuals in laboratory settings using fixed equipment, such as depth cameras. One example is the system developed by A. Dubois et al. [147], which utilized an RGB-D camera to assess fall risk. Through image processing algorithms, the trajectory of the human center of mass was estimated, enabling the extraction of gait parameters like step length, step duration, and gait velocity. The estimation error for these parameters was verified to be less than 6.6% when compared to a commercial altimetric carpet (GAITRite). While the depth camera-based system provides reliable sensing capabilities, it is cumbersome and highly dependent on the specific venue’s constraints.

To enhance the convenience of the monitoring process, H. A. Ghaida et al. [148] introduced a wearable insole equipped with three FSR sensors to monitor the CoP during a standing posture. Experimental results demonstrated a clear correlation between the developed insole and the F-Scan insole system (Tekscan, Norwood, MA, USA), which is commonly used by podiatrists [148]. The average root mean square (RMS) error of the total CoP measurement was 3 mm in the mediolateral direction and 2 mm in the anteroposterior direction.

In another study, D. Chen et al. [149] proposed a smart insole with a 96-piezoresistive sensor array and an IMU to classify different types of falls in the elderly. Parameters such as maximum ground reaction force difference, foot contact pitch, double-support phase duration, and pressure threshold-crossing point number were used to identify fall risks due to their close relationship with changes in body balance. With the aid of an SVM classifier, two types of falling events, namely slip and trip, could be recognized with an accuracy of 98.1%. L. Wang et al. [150] developed a smart insole using a three-axis accelerometer and a three-axis gyrosCoPe to detect falling events in the daily activities of older adults. A one-dimensional convolutional neural network model was employed to process the raw sensor data, achieving an accuracy of 98.61%. Compared to traditional fall hazard identification methods that rely on cameras, insole-based systems offer greater convenience and lower costs.

Apart from identifying falling events during walking, insole-based systems have also been utilized for predicting falling risks. For example, J. C. Ayena et al. [151] conducted a one-leg standing test for assessing a falling risk based on four FSR sensors placed at the heel and toes areas. The changes in the center of pressure (CoP) line were calculated during the experiments and compared with the participants’ self-assessment of falling risk using the Likert scale. The results indicated that the swing of the CoP is an effective indicator for evaluating the falling risk in the elderly.

Although various smart insole sensors have been proposed to detect and prevent accidental falls, there are still several challenges hindering their practical applications. The first challenge is comfort. While the insole sensors themselves can now be thin and flexible, the circuit board, battery, and wireless module still occupy a considerable volume, causing discomfort for the user. Secondly, the issue of energy consumption cannot be ignored. Insole sensors and data transmission require a constant energy supply for long-term daily monitoring, but current battery technology struggles to meet this demand. With the development of flexible circuits and advanced energy management, these problems are expected to be resolved in the near future, allowing insole-based systems to be widely employed for fall detection.

### 6.8. Brief Conclusion of Section 6

In summary, when designing an insole system for specific diseases, several considerations need to be taken into account, including abnormal gait features, sensing techniques, desired parameters, and detecting regions. The relevant information has been summarized in Table 9 for easy reference.

In terms of gait features and desired parameters, peak plantar pressure is closely associated with all six diseases, while shear pressure is specifically related to conditions that affect the movement of the CoP, such as flat foot, knee osteoarthritis (KOA), and falling events. The length of the gait phase is an important indicator for PD, stroke, and KOA. Additionally, diabetic foot and flat foot have specific indicators such as humidity, temperature, and arch height.

The selection of detecting regions is determined by the desired parameters. Although many products currently enable full-area detection, analyzing specific regions can provide more targeted results.

In terms of sensing techniques, piezoresistive sensors are the most suitable option, but piezoelectric sensors are also necessary for measuring shear pressure. Furthermore, the incorporation of cameras and inertial sensors can contribute to capturing integrated movements and models.

## 7. Challenges and Outlook

### 7.1. Challenges

#### 7.1.1. Black-Box Issue in Disease Diagnosis

To achieve high levels of accuracy in disease detection using machine learning algorithms, there is a strong demand for a comprehensive and extensive database. While such a database is currently unavailable, researchers from various institutions have created small-scale databases for specific disease diagnoses, and promising results have been reported, as discussed in previous sections.

However, the output results from machine learning models cannot yet be directly utilized for disease analysis. One of the main reasons for this is that the decision-making process of AI algorithms, often referred to as the “black-box” issue, is not fully understood by humans.

The black-box issue poses a significant challenge to the development of machine learning-supported disease analysis, particularly in the context of the IoHT where diagnosis data are transmitted to remote medical professionals without clear explanations of how the results are generated. For example, the PSD system can predict the occurrence of FoG by machine learning algorithms, but the explanation of prediction is not visible. Hence, the result can only assist a medical diagnosis because of the lack of explanation.

Unlike domains such as autonomous driving, where machine learning results can be directly used to make decisions (e.g., speeding up or performing a U-turn), medical data requires a comprehensive understanding before further treatments can be administered. As a result, current machine learning techniques are primarily utilized as assistive tools, limiting their effectiveness in addressing nonlinear and complex issues such as diagnosing diseases based on gait features.

In summary, the current dilemma involves the black-box issue, the lack of a large-scale database, and the constrained development of IoHT applications.

#### 7.1.2. Algorithms Misunderstand the Deviation of Gait Data

There is another dilemma associated with gait parameters. This dilemma relates to the potential bias that can arise during algorithm training due to the nature of gait parameters as a subset of biological data.

One of the contributing factors to this bias is the prevalence of duplicate or near-duplicate samples in gait parameter data [152]. Since gait parameters are derived from similar daily activities, the occurrence of duplicate samples is more apparent. Moreover, certain changes in parameters may not be immediately evident within a short period of time. When constructing a database for machine learning, duplicate samples often appear in both the training and testing datasets.

To simplify, when subtle changes in gait parameters are detected, although they may symbolize potential chronic diseases, the training algorithms may regard them as a normal fluctuation of gait data. Hence these subtle but crucial changes are overlooked by the training algorithms. The accuracy of diagnosis may therefore be influenced.

This bias in algorithm training presents a significant challenge when using gait parameters for disease diagnosis and analysis. Addressing this issue will require careful consideration and development of strategies to mitigate the impact of duplicate samples and ensure that the algorithms are capable of capturing the subtle variations indicative of specific chronic diseases.

In essence, while algorithms are effective in training machine learning models, they may not properly reflect the influence of neglected features. Consequently, the algorithms can inadvertently introduce bias based on the extracted features, leading to a decrease in the accuracy of rehabilitation and/or diagnosis evaluation [153].

#### 7.1.3. Hardware Decreases the Sensing Accuracy

The plantar pressure sensor, as a human-machine system for assisting medical diagnosis, requires its software to analyze daily gait data and provide references for medical treatment. Therefore, the accuracy of the data collected on the hardware side will significantly affect the effectiveness of the analysis.

However, there are several factors with the current foot-pressure sensor hardware that contribute to inaccurate data collection.

For instance, traditional resistive sensing technology is unable to detect shear stress [9], and piezoelectric sensing technology, due to the presence of piezoelectric effect in different directions [9], reduces the accuracy of detecting vertical pressure. Moreover, the sensor itself has noise, temperature drift, and zero drift, and there is relative slippage between the user’s foot and the insole, all of which decrease the accuracy of the collected data [46].

The above-mentioned problems will generate redundant data and errors, not only increasing the computational burden on the software side but also reducing the accuracy of data analysis. Therefore, there is an urgent need for sensors with more precise detection capabilities and the ability to calibrate errors more effectively.

### 7.2. Outlook

In the foreseeable future, we consider that the insole systems can be combined with the following trends to enhance their performance in assisting diagnosis and rehabilitation.

#### 7.2.1. Predict Patient’s Performance under Different Scenarios

For the purpose of enhancing performance in daily lives, patients are required to perform specific tasks during the rehabilitation training process. For example, analyzing the distribution of plantar pressure generated from walking on a flat surface can assess the progress of rehabilitation. However, these specific tasks may not cover all scenarios encountered in daily activities.

Therefore, it would be highly beneficial to develop methods that can use limited gait data to predict patients’ behavior in most, if not all, daily activities. For instance, algorithms can utilize gait data from walking on a smooth surface to predict performance on uneven terrain. Similar algorithms have been developed for applications such as Phase-Functioned Neural Networks for Character Control [154] and Local Motion Phases for Learning Multi-Contact Character Movements [155]. These examples highlight the potential for developing algorithms that can leverage gait data for a wide range of scenarios.

By developing such algorithms, it becomes possible to predict and analyze patients’ performance in various daily activities, providing valuable insights for rehabilitation and facilitating personalized treatment plans. This can contribute to improved outcomes and a more effective integration of rehabilitation into patients’ everyday lives.

#### 7.2.2. Multi-Sensing-Based Human Body Digital Twin (Hardware)

Digital twin (DT) technology involves creating a virtual replica of an object or system. This technology combines various sensors and sophisticated algorithms to analyze historical and real-time data gathered from these sensors. It has been shown that DT technology is effective in monitoring current performance and predicting behavior under different conditions. To build a comprehensive DT model, different types of sensors can be utilized, categorized into three types based on their application areas: muscle, orthopedic limbs, and neurons.

Therefore, the digital twin model (DT) can promote the development of plantar force sensors both in hardware and software.

As for hardware, the multi-sensor fusion utilizes various sensors to monitor daily data from different parts of the human body and uses this data to construct the user’s digital twin model.

For instance, IMU sensors can measure parameters such as gait velocity and ankle rotation, which are closely associated with lower limb movement. Electromyogram (EMG) or force myography (FMG) sensors can detect acceleration and posture, providing insights into the status of muscles [156]. Additionally, micro-electrode arrays (MEA) can capture changes in neurons by recording extracellular field potential.

The hardware of a multi-sensor system can compensate for the errors generated during data monitoring by capturing data from relevant parts of the user comprehensively. This is because data collected from different sensing technologies can be used for mutual calibration of the data.

#### 7.2.3. Multi-Sensing-Based Human Body Digital Twin (Software)

As for software, currently, wearable devices collect objective data. However, in many cases, the most effective solution to human chronic disease problems is actually relatively subjective, such as surveys, focus groups, interviews, and other methods based on behavioral science theories. Therefore, the establishment of DT provides a large amount of foundational data, which helps integrate the collection of subjective and objective data and medical analysis.

Meanwhile, by utilizing databases containing data on gait features, lower limb postures, and muscle activity, foot and plantar parts of DT models can be established. These models can assist medical professionals in predicting and estimating potential disease development trends, thereby enhancing the accuracy of diagnosis.

Overall, DT technology, with its integration of sensors and data analysis, offers significant potential for monitoring and predicting the behavior of objects or systems. In the context of healthcare, DT models can provide valuable insights for diagnosis and enable medical professionals to make informed decisions based on accurate predictions.

**Figure 7:**
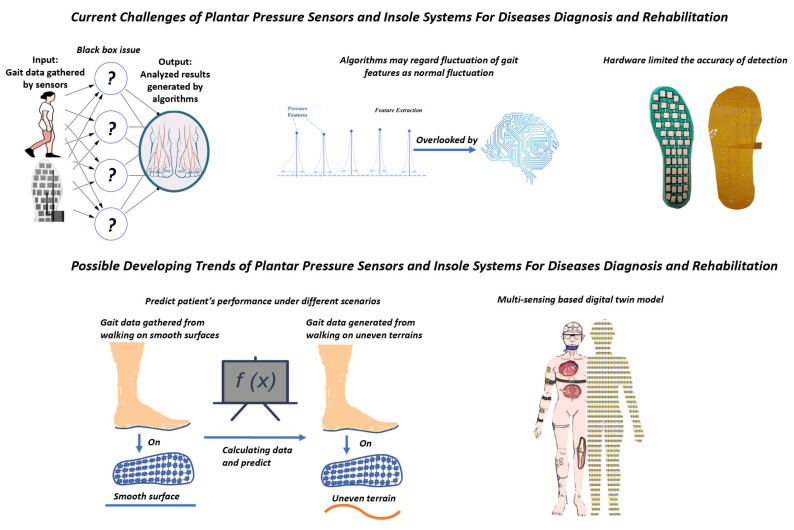
Summarization of challenges (including black-box issue, algorithms overlook gait data fluctuation, and limited accuracy due to hardware in Section 6) and outlook (including predict users’ performance under different scenarios and multi-sensing based digital twin model in Section 7).

Figure 7 comprehensively summarizes the challenges and outlook in Section 6 and Section 7, respectively.

## 8. Conclusions

In this essay, we have covered various aspects related to the emergence and application of insole systems for disease diagnosis and rehabilitation. This essay is structured as follows:

First, we provide a background on the development of insole systems and their significance in medical analysis in Section 1. We emphasize the role of gait features in diagnosing chronic lesions and monitoring patient rehabilitation.

It is important to introduce different sensing techniques of insole systems. Therefore, in Section 2, we discuss common sensing techniques used in gait analysis, including their mechanisms, detecting parameters, drawbacks, and advantages.

Since the above sensing techniques focus on obtaining gait data, we need to explain the stages of processing gait data. Hence, in Section 3, we present the three steps involved in processing gait data, namely data reconstruction, feature extraction, and data normalization. In Section 4 and Section 5, we review frequently used methods and their applications in each step.

After reviewing sensing techniques and gait data pre-processing methods, we then review the medical application of PSD sensors and insole systems. Therefore, in Section 6, we explain the mechanisms and abnormal gait features associated with six typical chronic diseases (PD, KOA, diabetic foot, flat foot, and stroke). Additionally, we review the corresponding insole systems designed for diagnosing and rehabilitating these diseases, establishing a connection between insole systems and diseases. We include diagrams illustrating abnormal gait features.

To analyze the current status of PSD sensors and insole systems more deeply, we discuss the present challenges and future trends, respectively.

In Section 7.1, we analyze the challenges faced by insole systems, focusing on gait parameters, the black-box issue, and hardware factors that decrease the sensing accuracy. These obstacles hinder the further development of insole systems by neglecting subtle changes and reducing interpretability and sensing accuracy.

In Section 7.2, we propose future developing trends of insole systems. The first one is improving algorithms to utilize a limited database to predict users’ gait parameters under different scenarios. The second one is related to hardware and software. By utilizing multi-sensing methods, such as combining EMG, IMU, and FMG sensors with PSD sensors, more detailed daily data of users can be obtained. Therefore, DT models of users can be established to assist medical professionals in diagnosing and analyzing.

Overall, this essay provides a comprehensive overview of insole systems, their application in medical analysis, the challenges they face, and potential solutions for future development.

## Figures and Tables

**Table 1:** Brief information of pressure reconstruction research in Section 3 [19,38,39,40,41,42,43,44,45,46].

Reconstructing Types	Techniques /Models	Result and Evaluation
Spatial Fitting [19]	Postprocessing algorithm is used to calculate perpendicular and shear stress amplitudes	Normal stress sensitivity: 56 mN Shear stress sensitivity: 173 mN
Spatial Fitting [38]	SCPM as model; GWR as function	RMSE of peak pressure <20 kPa Number of sensors >7
Temporal Fitting [39]	Divided insole into 6 regions; interpolating pressure data according to temporally adjacent average pressure	Calculated: peak pressure, pressure time integral, and center of pressure.
Fitting [40]	Values of four FSRs are used to calculate the resistance in x, y, z axes through fitting equations	Sensitivity: 375 kPa/V Sensing range: 0–800 kPa
Compressive Sensing [41]	Stage 1: OMP Stage 2: LASSO	RMSE of peak pressure <6.7 kPa When number of sensors = 4 Larger sensors produced lower RMSE
Compressive Sensing [42]	Stage 1: obtain separate PSD images Stage 2: Topelitz measurement matrix	Reconstructing accuracy: 97.76%
Compressive Sensing [43]	Gaussian Mixture Model (GMM) is used to reconstruct PSD map. Least Squares Model is used to estimate the value of pressure. Continuous plantar pressure map	When the K of GMM equals to 10 RMSE of pressure < 14 kpa
Compressive Sensing [44]	ℓdp- regularized least-squares (RLS) algorithm	Structural similarity index measure (SSIM) = 0.94841
Machine Learning [45]	Hardware: AMSA Algorithm: Support Vector Machine	Reconstructing results can make accuracy of classifying five body motions at 99.2%.
Machine Learning [46]	Support Vector Machine (SVM) Artificial Neural Network (ANN)	Reconstructed plantar pressure is used to classify gait phases and sub-phases Classification accuracy: 95.24% for stance-swing classification 87.08% for multi-phase classification

**Table 2:** Brief information of research of Section 4 [55,56,57,58,59,60,61,62,63,64,65,66,67].

Extracting Methods	Sensors/Techniques /Models	Features Extracted	Result and Evaluation
Fourier Transform [55]	DFT	Freezing of Gait (FOG)	Contributed to FOG diagnosis
Fourier Transform [56]	FFT and DFT	Freezing of Gait (FOG) Freeze Index (FI)	Contributed to FOG diagnosis
Fourier Transform [56]	Wavelet (WT, *n* = 14) and FFT (*n* = 8)	Pressure features like: Peak plantar pressure	Calculated 457 features
Peak detection [58]	Direct measurement	Peak plantar pressure	RMSE of pressure < 2.5 kPa
Peak detection [59]	3-D insole graphic visualization, LSTM	Peak pressure, cadence time, and stance ratio	Calculated temporal features (e.g., cadence)
Threshold division [60]	Digital images for threshold segmentation	Stride time, swing time, and velocity	RMSE of stride time, swing time, velocity are: 0.017, 0.019, 1.74, respectively
Threshold division [61]	The force of sensing points is decided by comparing output voltage to threshold voltage value (0.2 V)	Center of pressure (COP) Ground reaction force: shear and vertical	Calculated CoP through Equation (1)
Weight average [62]	Weighted average method	COP	RMSE of COP <13.8 mm
Weight average [63]	88 piezoresistive ink force sensors	COP in the direction of X and Y	RMSE in X direction <4 mm RMSE in Y direction is <10 mm
Summarization [64]	An insole system with 16 sensors distributed in a 4 × 4 matrix	Ground reaction force	Relative error of linearity: 5%, Hysteresis < 7.5%
Summarization [65]	Three-axis GRF measuring insole Silicone as sensing material Equation (2) is the model	Ground reaction force	The mean error < 10.7 N When shear pressure was 68.7 N
Machine learning [55]	ResNet, DFT, Transformer	Insole temperature	Calculated insole temperature with accuracy at 100%, 97.06%, 88.24%, respectively.
Machine learning [66]	GPR model and L5S1	Hip angle, knee angle, ankle angle, and lumbosacral joint angle	RMSE of X-axis and Y-axis were 0.21° and 0.22°, respectively
Machine learning [67]	PCA	Classify walking, descending, running, and falling (back, front, left, right)	Reduced number of features to a manageable level (18) Overall accuracy: 86%.

**Table 4:** Gait parameters of PD patients and healthy people [87,88,89].

Parameters	Patients	Healthy People
Gait velocity (m/s)		Higher
Gait cycle (s/step)	Higher	
Stride length (m)		Higher
Stride phase duration (s)	Higher	
Stance phase duration (s)	Higher	
Double support phase duration (s)	Higher	
Peak plantar pressure (kPa)		Higher
Plantar pressure central frequency (Hz)	Higher	

**Table 5:** Typical gait patterns of stroke patients [117,118].

Gait features	Affected Lower Extremity	Unaffected Lower Extremity
Swing phase (%)	-	higher
Stance phase (%)	higher	-
Single support phase (%)	higher	-
Double support phase (%)	higher	-
Ground reaction force (N)	-	higher
Peak pressure (kPa)	-	higher

**Table 6:** Parameters of flat foot diagnosis [125,126,127,128].

Parameters	Description	Flatfoot Patients
Arch height	Maximum arch distance from the ground	<1 cm
Chippaux-Smirak index (C-S index)	Ratio between the minimum arch width and the maximum forefoot width	>45%
Barkhusen index	Ratio between contact and non-contact area of plantar	>2
CoP excursion index (CPEI)	Ratio between CoP deviation toward the lateral foot and foot width	<14%
Ankle rotation angle	In both sagittal plane and coronal plane	Higher internal rotation, higher plantarflexion angle, lower dorsiflexion angle a)
Stance phase duration	/	Lower in the early stance phase a)

**Table 7:** Critical indicators of KOA patients [134,137,138].

Parameters	Patients with KOA	Health People
Forefoot pressure transfer mode	Dispersing between the medial and the center of the forefoot	First load the central part and then move to the medial part
Single support phase duration	-	Longer
Anteroposterior length of CoP path	-	Higher
Transverse width of CoP path	-	Higher
Peak value of KAM	Higher	-

**Table 8:** Explanation of gait parameters related to falling. [79,80,81,145,146].

Gait Parameters	Explanation
Swing phase	Duration of time that a foot spends in the swinging motion
Stance phase	Duration of time that a foot remains in contact with ground
Stride length	The distance between two consecutive foot contacts of the same foot
Step length	The distance between the points where the two feet make contact with the ground
Peak plantar pressure	The maximum pressure of the foot on the insole
Center of pressure (COP)	The pressure center of a single foot during walking or standing, while total CoP displacement emphasizes the pressure center of both feet in the standing posture
Velocity	The rate of changing of position with respect to time

**Table 9:** Main abnormal gait features, detection sites, detection parameters for each disease.

Diseases	Main Abnormal Gait Features	Sensing Techniques	Desired Parameters	Suitable Detecting Regions
Parkinson’s disease (PD)	Lower peak plantar pressure Longer gait periods Lower step length	Inertial sensors Piezoresistive sensors Resistive sensors	Phase duration Peak plantar pressure Gait velocity	Heel First metatarsal
Diabetic foot	Higher insole temperature Higher peak plantar pressure Higher insole humidity	Piezoresistive sensors Capacitor sensors Thermal-humidity	Temperature Humidity Peak plantar pressure	Toes Arch Heel
Stroke	Shorter swing phase Longer stance phase Less shear plantar pressure Less normal plantar pressure	Piezoresistive sensors Piezoelectric sensors	Peak plantar pressure Shear plantar pressure Phase duration	Whole area detection
Flat foot	Less arch height Larger ankle rotation Shorter stance phase	Piezoresistive sensors Photo capturing	Rotation angle Arch height Gait cycles	Heel Arch First metatarsal
Knee osteoarthritis (KOA)	Longer single-support stance phase Higher peak plantar pressure	Piezoresistive sensors Piezoelectric sensors (potential)	Peak plantar pressure Phase duration CoP path length and direction	Whole area is the most suitable; Center of foot Heel
Elderly falling	Abnormal gait velocity Shear plantar pressure deviation CoP movement	Piezoresistive sensors Piezoelectric sensors (potential)	Shear plantar pressure CoP’s position Double-support phase duration	Toes Heel Center of foot

## Data Availability

Not applicable.
